# Development and Evaluation of a Hybrid Measurement System to Determine the Kinematics of the Wrist

**DOI:** 10.3390/s24082543

**Published:** 2024-04-16

**Authors:** Jason Dellai, Martine A. Gilles, Olivier Remy, Laurent Claudon, Gilles Dietrich

**Affiliations:** 1 Institut National de Recherche et de Sécurité (INRS), 54519 Vandoeuvre-lès-Nancy, France; martine.gilles@inrs.fr (M.A.G.); olivier.remy@inrs.fr (O.R.); laurent.claudon@inrs.fr (L.C.); 2 Institut des Sciences du Sport Santé de Paris (URP 3625), Université Paris Cité, 75015 Paris, France; gilles.dietrich@u-paris.fr

**Keywords:** MIMU, optical motion capture system, accuracy, wrist kinematic, hybrid system

## Abstract

Optical Motion Capture Systems (OMCSs) are considered the gold standard for kinematic measurement of human movements. However, in situations such as measuring wrist kinematics during a hairdressing activity, markers can be obscured, resulting in a loss of data. Other measurement methods based on non-optical data can be considered, such as magneto-inertial measurement units (MIMUs). Their accuracy is generally lower than that of an OMCS. In this context, it may be worth considering a hybrid system [MIMU + OMCS] to take advantage of OMCS accuracy while limiting occultation problems. The aim of this work was (1) to propose a methodology for coupling a low-cost MIMU (BNO055) to an OMCS in order to evaluate wrist kinematics, and then (2) to evaluate the accuracy of this hybrid system [MIMU + OMCS] during a simple hairdressing gesture. During hair cutting gestures, the root mean square error compared with the OMCS was 4.53° (1.45°) for flexion/extension, 5.07° (1.30°) for adduction/abduction, and 3.65° (1.19°) for pronation/supination. During combing gestures, they were significantly higher, but remained below 10°. In conclusion, this system allows for maintaining wrist kinematics in case of the loss of hand markers while preserving an acceptable level of precision (<10°) for ergonomic measurement or entertainment purposes.

## 1. Introduction

The analysis of wrist kinematics is key to gesture execution studies. This analysis is crucial for numerous practical applications, whether for estimating musculoskeletal disorders (MSDs) in the workplace [1,2,3] or understanding sport movements [4,5,6]. When it comes to laboratory research, the gold standard technique for 3D kinematic analysis involves the use of optoelectronic systems or Optical Motion Capture Systems (OMCSs). These optical systems use high-resolution and high-frequency infrared cameras that register the movements of passive self-reflective markers placed on the subject. Three non-collinear markers are needed to determine the orientation of a segment in space. Consequently, to determine wrist kinematics, an OMCS records the movements of reflective markers attached to a hand, a forearm, and possibly the upper arm to determine the orientation of these segments and calculate joint angles [7]. During the evaluation of wrist kinematics, especially in a professional context, problems frequently arise when it comes to detecting the markers that can be occluded, whether by other body parts or nearby objects. These occlusions can often be observed during the wrist kinematic evaluation with hairdressers during their professional activities as a result of repetitive hand movements through hair. In that context, it can be interesting to use other sensors that rely on non-optical sources of information, such as magneto-inertial units (MIMUs).

MIMUs allow us to precisely determine the orientation of a segment in space. They consist of an accelerometer, a gyroscope, and a magnetometer. The data issued from these various sensors are then integrated using a fusion algorithm that provides orientation. Due to the way they operate, MIMUs enable measurements to be taken in broader contexts than the laboratory. For example, they were used to determine the orientation of a tennis racket in learning situations [8], to investigate whole body kinematics adaptations when running on different surfaces [9], to evaluate knee kinematics during a home rehabilitation [10], and also to monitor the activities of palm oil workers [11]. The autonomy of these devices placed directly on the skin is what makes them easily transportable and usable on-site. However, they also have limitations. To begin with, they are less precise than an Optical Motion Capture System (OMCS). Bergamini et al. [12] compared the measurements from a MIMU and an OMCS for hand orientation in everyday gestures. They observed average errors expressed in root mean square errors (RMSEs) of around 5° to 10° per axis of rotation. Systems that use several MIMUs have similar or even more significant errors. For example, by measuring shoulder angles during handling tasks, Poitras et al. [13] observed average errors of 12° per joint angle. Similarly, during dynamic movements involving the entire body, Mavor et al. [14] observed average errors from 4° to 40° depending on the observed joint angle. Nevertheless, the average errors were around 9°. In addition to that, MIMUs can be affected by temporal drift, given that they integrate linear angle accelerations [15]. Furthermore, magnetometer measurements can be affected by various magnetic fields present in the close environment of these sensors [16].

Nowadays, many companies have their own entry-level MIMU offering, among which Bosch Sensortec^®^ (BST) (Reutlingen, Germany), STMicroelectronics^®^ (Geneva, Switzerland), Digilent^®^ (subsidiary of National Instrument^®^, Austin, TX, USA), and Invensens^®^ (subsidiary of TDK Electronics^®^, Munich, Germany) can be mentioned. These groups offer numerous MIMUs that vary in characteristics, size, and price. Although they were not initially intended for movement analysis, these MIMUs are beginning to be used in various studies on human movement. Indeed, they allow for more modularity than some other systems designed for movement analysis. For example, MIMUs of this type have been used to determine the range of a shoulder joint [17], the orientation of a table tennis racket in space [18], and even hand orientation when hitting a baseball [19]. However, even though this equipment might seem interesting owing to its low price and high modularity, the accuracy of each sensor needs to be evaluated before its use in the research environment. This step is of the essence, especially given that the range of sensors is vast and diverse, and this technology is in constant and fast-paced development.

In order to benefit from both techniques (MIMUs and OMCSs), a hybrid measurement system combining an OMCS for proximal body segments and a MIMU for hand orientation evaluation has been considered. This approach allows us to maintain a high level of accuracy for less occluded segments while maintaining maximum hand orientation, which is the segment with the highest risk of occlusion, although with less accuracy. This type of hybrid device that combines an OMCS and inertial sensor has already been proposed in the past. For instance, Roetenberg and Veltink [20] used an inertial measurement unit in addition to passive markers to maintain the orientation of an object in space during marker occlusion. However, coupling data from an OMCS and a MIMU requires the synchronization of both systems in time and expression of orientation in the same frame of reference. High-end motion analysis systems using MIMUs usually integrate these functionalities. In cases where only entry-level MIMUs are integrated, it is necessary to do several calculations so that both tools share the same frame of reference.

The objective of this article is to present the development and evaluation of the accuracy of a hybrid system for wrist kinematics measurement combining an OMCS for forearm orientation measurement and a MIMU for hand orientation measurement. This work required two phases. (1) A development phase, whose aim was to integrate the orientation of an entry-level MIMU into an OMCS. (2) An application phase, evaluating the accuracy of the hybrid system in determining wrist kinematics. The selected application case refers to the analysis of professional gestures in the field of hairdressing, which is characterized by a high risk of marker occlusion.

## 2. Materials and Methods

### 2.1. Device Description

The MIMU used in this research was the BNO055 (Bosch Sensortec^®^, 2013) version 3.14 integrated into the ADA2472 module by Adafruit^®^ (New York, NY, USA). This MIMU was selected due to its ability of automatic calibration and the fact that it possesses its own fusion algorithm: the BSX developed by Bosch Sensortec^®^. It provides orientation in the form of quaternions at 50 Hz in a magneto-inertial reference frame. This MIMU has already been used in various studies on human movement [17,18,21]. However, no study has tried to evaluate its accuracy in determining an orientation relative to an OMCS.

**Figure 1:**
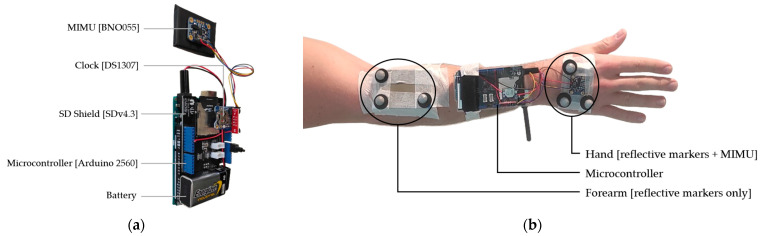
Detailed view of the different components (**a**). Positioning of the MIMU device and markers on the right upper limb (**b**): The microcontroller was attached to the forearm using a Velcro strap and a reusable hose clamp. The plate supporting the hand markers and the MIMU was placed on the distal part of the second and third metacarpals to accurately capture the orientation of the back of the hand. Furthermore, it was verified that the axes of the MIMU and the cluster of points were indeed parallel. The markers attached to the hand were independent of the hybrid system [MIMU + OMCS]. These markers were used to assess the accuracy of the hybrid system compared to a measurement performed entirely with the OMCS.

To control the MIMU, an Arduino^®^ (Somerville, MA, USA) 2560 controller with an ATmega 2560 microprocessor clocked at 16 MHz was used. An I2C bus made communication possible, and a clock (DS1307, Adafruit^®^) was used to timestamp the files (Figure 1a). Data from the accelerometer, gyroscope, magnetometer, and orientation as well as the calibration index were written in ASCII format to an SD card using an SD Shield (SD v4.3, Seeed Studio^®^ (San Francisco, CA, USA), 2014). The timestamping of the data was performed using the ‘micros()’function of the Arduino. The data were written at a sampling frequency ranging between 100 and 300 Hz. Data coinciding with the 50 Hz timestamp were then retrieved.

For its evaluation, the MIMU was coupled with an OMCS as a reference tool. The OMCS used was the Motion Analysis^®^ (Rohnert Park, CA, USA) system consisting of 10 Eagle Hawk II cameras. The data were registered at a frequency of 50Hz using the Cortex 9.1.0.8^®^ software. The optoelectronic system was calibrated according to the manufacturer’s recommendations. Calibration was carried out using a 500 mm calibration wand. Generally speaking, optoelectronic systems have errors of <1 mm [22].

The orientation of the hand was simultaneously determined with the use of both systems (OMCS and [MIMU + OMCS]). The MIMU, with three reflecting markers, was placed on a plate attached to the back of the hand (Figure 1b). The orientation of the forearm was evaluated only by the OMCS using three markers placed in right triangles, also attached to the plate. The plate was placed on the proximal part of the forearm. This configuration allowed us to account for wrist angles (flexion/extension and adduction/abduction), as well as the pronation–supination of the upper limb [7]. Motion Inspector^®^ software (v1.92) was used to determine the quaternions representing the orientation of each of the two segments in the OMCS reference frame.

### 2.2. Integration of MIMU into an OMCS

Quaternions allow us to represent an orientation in 3D using 4 parameters [23]. Thus, singularities, such as gimbal lock associated with Euler angles, can be avoided. Quaternions are hypercomplex numbers that can be written in the following way (Equation (1)):

Q = a + bi + cj + dk(1)

Quaternions Q have a real part (a) and an imaginary part (bi + cj + dk, where i, j, k are imaginary units). Quaternions representing orientation are called versors, and they have a norm equal to 1. 

In order to introduce the orientation provided by a MIMU into an OMCS, it is necessary for both systems to express orientations in the same reference frame, in addition to being synchronized in time. Generally, MIMU devices will express the data in a magneto-inertial (or global) reference frame, which allows the expression of relative orientation between two MIMUs. In order to switch from a global (or magneto-inertial) reference frame to a frame attached to a MIMU, it is necessary to perform a “heading reset”. This operation involves setting the orientation of three axes to zero (Equations (2) and (3)).

Q_offset_ = Q_ref_ ⊗ Q_initial_MIMU_global_, (2)

where Q_offset_ represents a rotation between the orientation of the object in its reference frame and the orientation of the object in the global reference. It corresponds to the product (⊗) of twoquaternions: Q_ref_, a unit quaternion without rotation such as Q_ref_ = [1 0 0 0], and Q_intial_MIMU_global_, the initial orientation of the object given by the MIMU in the global frame.

Q_data_MIMU_stradown_ = Q_offset_^−1^ ⊗ Q_data_MIMU_global_, (3)

where Q_data_MIMU_Stradown_ is the orientation determined by the MIMU of the object in the object’s reference frame. It is the product of Q_data_MIMU_global_, the orientation of the object determined by the MIMU in the global frame, and of Q_offset_^−1^, conjugate of Q_offset_.

The orientation of the MIMU in a reference frame attached to the object can be expressed in another reference frame. If the initial orientation of the object in the reference frame of the OMCS is known, a quaternion sandwich product (Equation (4)) enables the transition from the reference frame attached to the object to the reference frame of the OMCS.

Q_data_MIMU_OMCS_ = Q_initial_OMCS_ ⊗ Q_data_MIMU_stradown_ ⊗ Q_intial_OMCS_^−1^ (4)

where Q_data_MIMU_OMCS_ represents the orientation of the object determined by the MIMU in the reference frame of the OMCS, and Q_initial_OMCS_ represents the initial orientation of the object determined by the same system and in its reference frame.

This method means that the object or the studied body segment is equipped at the same time with a MIMU and reflective markers, enabling us to determine its orientation in the OMCS. Other methods allow carrying out this stage. For example, it is possible to place a square equipped with three markers indicating the north in the capture space of the OMCS. The orientation of this square allows for the transition from a global reference frame to that of the OMCS.

Moreover, during human movements, the relative orientation of a body segment in relation to another can be measured using quaternions (Equation (5)). Nevertheless, it is necessary to ensure that the rotational axes between segments match the joint rotation axes.

Q_relative_orientation_ = Q_proximal_segment_^−1^ ⊗ Q_distal_segment_ (5)

where Q_relative_orientation_ represents the relative orientation of the segment. It is the product of the conjugate of Q_proximal_segment_, the orientation of the proximal segment, and Q_distal_segment_, the orientation of the distal segment. The orientations of proximal and distal segments must be expressed in the same reference frame.

### 2.3. Determining Joint Angles

Initially, the quaternions of the hand and the forearm determined using reflective markers enabled the determination of the relative orientation of the hand to the forearm by the OMCS (Equation (5)). Secondly, the relative orientation of the hand to the forearm was determined using the hybrid system [MIMU + OMCS]. The quaternion representing the hand orientation calculated using reflective markers was replaced by the quaternion calculated by MIMU. The data from the MIMU were previously expressed in the reference frame of the OMCS (Equations (2)–(4)). At the beginning of each recording, the subject performed a maximum flexion of the wrist before returning to the reference position. By observing the absolute orientation of the hand determined by the MIMU and the OMCS, it was possible to manually synchronize the two systems.

**Figure 2:**
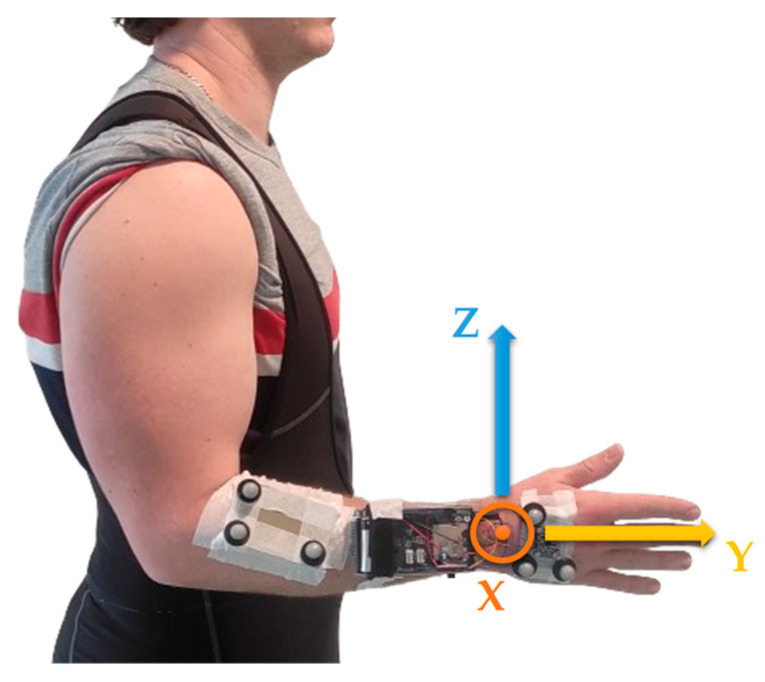
Neutral position of a wrist, arm alongside the body, elbow flexed at 90°, and a thumb pointing upwards [24]. Maintaining this position allows the defining of an offset related to the orientation of the two rigid plates when the wrist is in a neutral position. Rotation axes of the wrist are also indicated: the axes Z and X correspond to the flexion/extension and adduction/abduction axes. The Y axis corresponds to the axis of pronation/supination.

For the two methods (OMCS alone and the system [MIMU + OMCS]), the relative orientation of the hand to the forearm was reset by performing a heading reset (Equations (2) and (3)) as the subject had their wrist in a neutral position (Figure 2).

Finally, in order to ensure interpretable results in the context of human kinematic evaluation, the quaternions representing the relative orientation of the hand to the forearm were converted into Euler angles. The chosen sequence was ZXY (Figure 2), where the Z axis corresponds to flexion/extension, the X axis to adduction/abduction, and the Y axis to pronation/supination [24]. To retain maximum information, the kinematic data obtained from both systems were not filtered.

### 2.4. Experimental Setup

This study was part of preliminary work aimed at developing a technical solution for a future study. The accuracy of the hybrid system [MIMU + OMCS] was evaluated in the context of complex gesture analysis, namely those performed while cutting and styling hair. In order to compare the two systems, one subject (non-hairdresser) participated in the study [25,26]. Once placed on the right hand, the MIMU was calibrated based on the recommendations of the manufacturer. The subject reproduced two simple gestures that hairdressers typically perform: a straight cut (cutting gesture; CUT) and movements performed while combing (combing gesture; COMB) (Table 1). The straight cut consists of cutting the hair in a straight line horizontally. Combing gestures consist of hair straightening by arranging it in organized sections in the hand that will hold it for cutting. These two gestures that involve the right hand were performed for 50 s. Each gesture underwent 9 trials. The first and last 10 s of the gesture execution sequence were excluded from the comparison. The angular velocity of the wrist was also measured for each of the two gestures. 

### 2.5. Statistical Analysis 

The wrist and forearm angles, expressed in Euler angles, determined using the hybrid method [MIMU + OMCS], were compared to those obtained entirely with OMCS using the root mean square method (RMSE). The RMSE indicates the average absolute error between two measurement methods over time.

The results of the angular differences are presented in the form of mean values and standard deviations. ANOVAs (and *t*-tests) were used to compare the mean values. The significance threshold for the tests was set at 5%. The analysis was conducted using Stata^®^ software version 17.

## 3. Results

**Figure 3:**
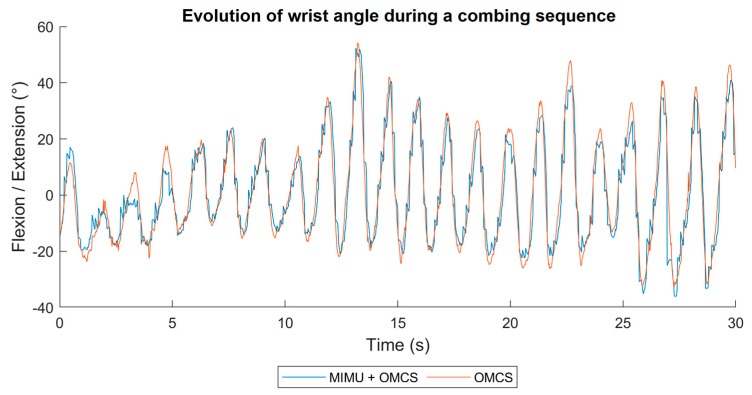
Evolution of the flexion/extension angle determined by the hybrid system [MIMU + OMCS] (in blue) and the OMCS (in orange) during the sequence in which the comb was used (COMB).

**Figure A1:**
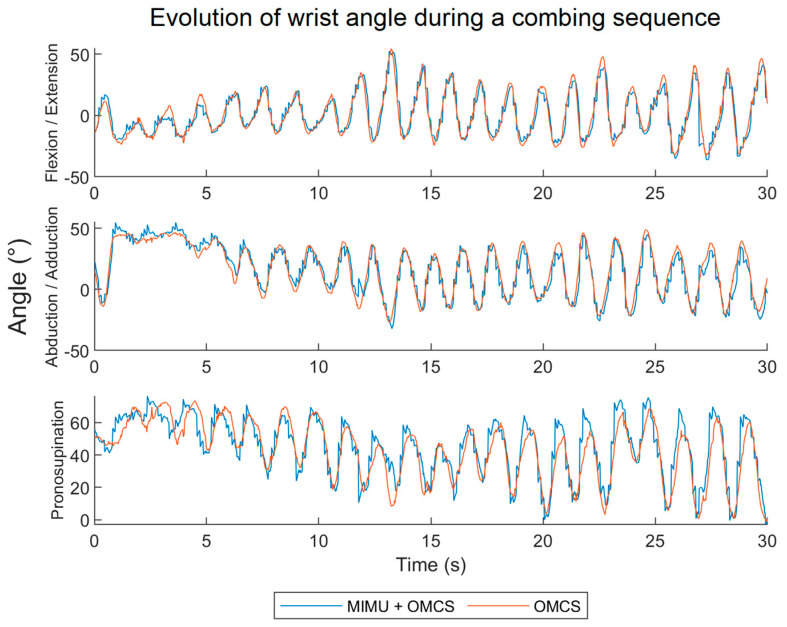
Evolution of flexion/extension, abduction/adduction, and pronation/supination angles determined with the hybrid system [MIMU + OMCS] (in blue) and with the OMCS (in orange) for a combing sequence.

**Figure A2:**
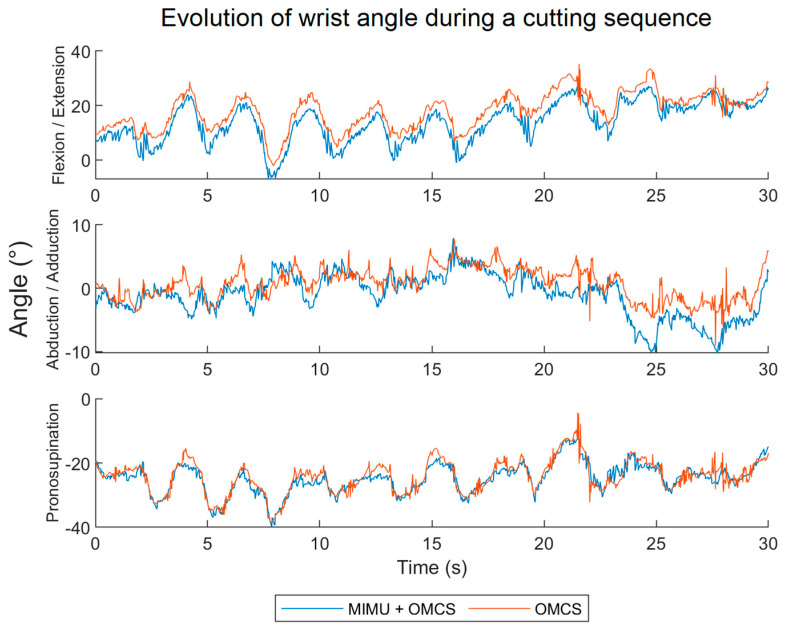
Evolution of flexion/extension, abduction/adduction, and pronation/supination angles determined with the hybrid system [MIMU + OMCS] (in blue) and with the OMCS (in orange) for a cutting sequence.

Figure 3 shows the evolution of the flexion/extension angle of the wrist joint, which was measured using the two systems during a COMB trial. The evolution of the adduction/abduction angle and pronosupination for the same sequence, as well as for the hair cutting sequence, are available in the Appendix A Figure A1 and Figure A2).

The results concerning the RMSE for the gestures performed during the hair cutting are indicated in Table 2. We observed significantly larger errors for each degree of freedom during COMB compared to CUT (p_flexion/extension_ = 0.043, p_adduction/abduction_ < 0.001, p_pronosupination_ < 0.001). The characteristics of the mean speed measured using the reference tool are indicated in Table 3. We observed higher mean speed during COMB (p_flexion/extension_ < 0.001, p_adduction/abduction_ < 0.001, p_pronosupination_ = 0.017).

## 4. Discussion

The objective of this work was to develop a hybrid measurement system [MIMU + OMCS] to evaluate wrist kinematics and assess its accuracy in simulated hair cutting situations. Two common professional gestures were studied (CUT and COMB). While performing the hair cutting gestures, RMSEs of 4.5° ± 1.5° for flexion/extension, 5.1° ± 1.3° for adduction/abduction, and 3.7° ± 1.2° for pronation/supination were observed. While performing combing gestures, larger RMSEs were observed: 5.8° ± 0.9° for flexion/extension, 8.5° ± 1.4° for adduction/abduction, and 8.5° ± 2.2° for pronation/supination. These results are consistent with those observed in various previous studies that evaluated the accuracy of the systems using one of several MIMUs for different movements relative to an OMCS. For instance, concerning simple trunk movements, a study evaluated the accuracy of a high-end MIMU system (Xsens^®^ and Noraxon^®^, Henderson, NV, USA) compared to an OMCS [27]. They observed RMSEs ranging from 3.2° to 7.4°, depending on the systems and angles considered. Another study [28] focused on the accuracy of an entry-level system (Perception Neuron^®^, Miami, FL, USA) and reported an RMSE of around 5°. Regarding upper limb movements, in a study [13] comparing a system using a MIMU (Xsens^®^) with an OMCS (Vicon^®^, Hauppauge, NY, USA), RMSEs of 6.7° for single-axis gestures and 11.5° during load manipulation tasks were observed. Similar observations of RMSEs between 5° and 10° have been reported in studies involving whole-body MIMU systems during load-carrying tasks [29]. A recent study [14] evaluated a MIMU-based system’s precision during dynamic movements (running, walking, squatting, lying down, etc.) performed by military personnel, with observed RMSEs ranging from 4° to 40° depending on the joint and degree of freedom considered. The authors concluded that this level of accuracy was satisfactory for observing gesture variability in a military context. 

Another study [19] evaluated the accuracy of a dual-MIMU system compared to an OMCS for assessing trunk and hand orientations during baseball bat manipulation. RMSEs lower than 5° in different planes were considered “excellent” and RMSEs lower than 10° as “good”. These threshold values have been used by other authors in determining the precision of a system for tracking activities in situ [30,31]. Applying these thresholds to the present study’s results, the precision during cutting gestures can be considered excellent for flexion/extension and pronation/supination, and good for adduction/abduction. Regarding combing gestures, the precision can be described as good. Moreover, the significantly larger errors observed during combing gestures may be explained by significantly higher angular velocities. Comparable observations between MIMU precision and angular velocity have been found previously [32]. This observation is particularly important in this evaluation as the performed gestures were executed by a non-expert in hairdressing. It is likely that professionals would perform these gestures at higher speeds, which could result in greater degradation of MIMU precision in real work situations. Additionally, the precision evaluation was conducted on relatively short recordings, whereas many MIMU systems drift over time [33]. The effect of longer recordings lasting several minutes on data quality was not considered.

The evaluated hybrid system appears to be suitable for ergonomic studies, such as analyzing daily activities or work situations [34]. Nevertheless, for more detailed biomechanical studies, especially regarding gesture variability, higher precision is required [35,36]. Movements of the upper limb during fine manipulations often exhibit variations below 5°. For example, in tasks like pointing [37], learning stone cutting [38], or assembling components [39], the measured variability of the involved joints was around 3°. Thus, the threshold defined as “excellent” by Punchihewa et al. [19], although appropriate for evaluating ergonomic tools and reflecting typical MIMU precision, is ultimately less suitable for fine biomechanical analysis.

Today, some OMCSs allow direct real-time integration of MIMU data into motion capture software. For example, the Nexus 2 (Vicon^®^, Hauppauge, NY, USA) enables the integration of Blue Trident MIMU (Vicon^®^, Hauppauge, NY, USA), while the NoitomVPS (Perception Neuron^®^, Miami, FL, USA) allows motion capture with hybrid Tracker (MIMU + markers). But this addition possibility is recent. Only few OMCSs possess this possibility, and not many MIMUs are currently compatible with it. Therefore, coupling a MIMU with an OMCS will, in most cases, need several calculation steps before the two tools share the same reference frame. Therefore, to ensure the highest possible accuracy of such hybrid systems, it is also possible to determine the orientation of the segments that might be occluded using both reflective markers and a MIMU at the same time and supplement the loss of markers by the OMCS with MIMU data [20]. This type of system has started developing the function to capture a movement in the entertainment sector. However, in the context of fine biomechanical analysis, having variable precision depending on occluded segments raises concerns.

Furthermore, even though the BNO055, available on the market since 2013, has been very popular and easy to use (auto-calibration and integrated fusion algorithm), there are more recent sensors. Given the constant technical progress in this field, the question of the accuracy of these sensors in assessing human kinematics remains to be investigated. Additionally, this study did not attempt to evaluate the precision improvement provided by a hybrid system compared to a solely MIMU-based system. It would have been interesting to include a comparison with a system using two MIMUs to assess the benefits offered by hybridization with an OMCS.

## 5. Conclusions

This article presents a methodology for integrating the orientation of an entry-level MIMU (BNO055) into an OMCS to determine wrist kinematics. The method outlined allows for the incorporation of any MIMU providing quaternions into an OMCS at a low material cost. This facilitates the determination of segment or object orientations that may be masked or difficult to equip with markers. In addition, this article proposes a concrete case of hybridization aimed at determining wrist angle based on orientations determined by a MIMU and an OMCS. The accuracy of the developed system is similar to that of certain systems dedicated to measuring human kinematics, entirely relying on MIMUs. Given the accuracy levels presented in the literature, this system can be used for human kinematic measurement in ergonomics or in the entertainment industry, particularly in situations where the risk of marker occlusion is high. However, this level of precision may be limiting in the context of detailed biomechanical analysis of movement. 

Based on this work, two areas of technical development are conceivable. Firstly, it is possible to improve the hybrid system [MIMU + OMCS] by introducing a more precise MIMU, reducing the size of the microcontroller, or integrating the possibility of having live data feedback. Improving these points would enable the system to be used for fine biomechanical analyses with a high risk of obscuring markers. In addition, it could be interesting to determine the real gain of such a system when the MIMU is used in addition to a marker cluster in a potentially occulting environment. Secondly, given its price and technical features (automatic calibration and on-line quaternion calculations), the precision of the BNO055 has proved to be an interesting sensor for measuring segment orientation outside the laboratory. By modifying the methodology presented in this article, it is possible to develop a system for measuring wrist kinematics using two BNO055s. It would then be possible to develop a low-cost, easily scalable motion capture system using several BNO055s and evaluate its accuracy compared with an optoelectronic system.

## Figures and Tables

**Table 1:** Organization of a trial.

Step	Time
Reference position	5 s
Maximum flexion	5 s
Reference position	5 s
Gesture (CUT or COMB)	50 s
Reference position	10 s

**Table 2:** RMSE during CUT and COMB. Among the 9 recordings, one of the comb recordings was not considered, as the MIMU showed aberrant values after 20 s.

RMSE (Degrees)	CUT (N = 9)	COMB (N = 8)
Flexion/Extension	4.5 ± 1.5	5.8 ± 0.9
Abduction/Adduction	5.1 ± 1.3	8.5 ± 1.4
Pronation/Supination	3.7 ± 1.2	8.5 ± 2.2

**Table 3:** Description of the angular speed during CUT and COMB.

Mean Speed (deg·s^−1^)	CUT (N = 9)	COMB (N = 8)
Flexion/Extension	39.1 ± 9.0	52.2 ± 11.1
Abduction/Adduction	23.0 ± 5.0	56.7 ± 10.5
Pronation/Supination	31.3 ± 9.5	77.1 ± 15.2

## Data Availability

The raw data supporting the conclusions of this article will be made available by the authors on request.

## References

[1] Weitbrecht M., Holzgreve F., Fraeulin L., Haenel J., Betz W., Erbe C., Maurer-Grubinger C., Wanke E.M., Brueggmann D., Nienhaus A. (2022). Ergonomic Risk Assessment of Oral and Maxillofacial Surgeons—RULA Applied to Objective Kinematic Data. Hum. Factors.

[2] Kato A.E., Fathallah F.A., Miles J.A., Meyers J.M., Faucett J., Janowitz I., Garcia E.G. (2006). Ergonomic Evaluation of Winegrape Trellis Systems Pruning Operation. J. Agric. Saf. Health.

[3] Wallius M.-A., Bragge T., Karjalainen P.A., Järvelin-Pasanen S., Rissanen S.M., Vartiainen P., Räsänen K. (2018). Effects of Mop Handle Height on Forearm Muscle Activity, Wrist and Upper Arm Posture and Movement During Floor Mopping. IISE Trans. Occup. Ergon. Hum. Factors.

[4] Busuttil N.A., Reid M., Connolly M., Dascombe B.J., Middleton K.J. (2022). A Kinematic Analysis of the Upper Limb during the Topspin Double-Handed Backhand Stroke in Tennis. Sports Biomech..

[5] Carson H.J., Richards J., Mazuquin B. (2019). Examining the Influence of Grip Type on Wrist and Club Head Kinematics during the Golf Swing: Benefits of a Local Co-Ordinate System. Eur. J. Sport Sci..

[6] Okubo H., Hubbard M. (2015). Kinematics of Arm Joint Motions in Basketball Shooting. Procedia Eng..

[7] Turner J., Forrester S.E., Mears A.C., Roberts J.R. (2020). The Influence of Tracking Marker Locations on Three-Dimensional Wrist Kinematics. J. Sci. Med. Sport..

[8] Williams B.K., Sanders R.H., Ryu J.H., Graham-Smith P., Sinclair P.J. (2021). Racket Orientation Angle Differences between Accurate and Inaccurate Squash Shots, as Determined by a Racket Embedded Magnetic-Inertial Measurement Unit. Sports Biomech..

[9] Mohr M., Peer L., De Michiel A., van Andel S., Federolf P. (2023). Whole-Body Kinematic Adaptations to Running on an Unstable, Irregular, and Compliant Surface. Sports Biomech..

[10] El Fezazi M., Achmamad A., Jbari A., Jilbab A. (2023). A Convenient Approach for Knee Kinematics Assessment Using Wearable Inertial Sensors during Home-Based Rehabilitation: Validation with an Optoelectronic System. Sci. Afr..

[11] Chan Y.S., Teo Y.X., Gouwanda D., Nurzaman S.G., Gopalai A.A., Thannirmalai S. (2022). Musculoskeletal Modelling and Simulation of Oil Palm Fresh Fruit Bunch Harvesting. Sci. Rep..

[12] Bergamini E., Ligorio G., Summa A., Vannozzi G., Cappozzo A., Sabatini A.M. (2014). Estimating Orientation Using Magnetic and Inertial Sensors and Different Sensor Fusion Approaches: Accuracy Assessment in Manual and Locomotion Tasks. Sensors.

[13] Poitras I., Bielmann M., Campeau-Lecours A., Mercier C., Bouyer L.J., Roy J.-S. (2019). Validity of Wearable Sensors at the Shoulder Joint: Combining Wireless Electromyography Sensors and Inertial Measurement Units to Perform Physical Workplace Assessments. Sensors.

[14] Mavor M.P., Ross G.B., Clouthier A.L., Karakolis T., Graham R.B. (2020). Validation of an IMU Suit for Military-Based Tasks. Sensors.

[15] Trojaniello D., Cereatti A., Pelosin E., Avanzino L., Mirelman A., Hausdorff J.M., Della Croce U. (2014). Estimation of Step-by-Step Spatio-Temporal Parameters of Normal and Impaired Gait Using Shank-Mounted Magneto-Inertial Sensors: Application to Elderly, Hemiparetic, Parkinsonian and Choreic Gait. J. NeuroEngineering Rehabil..

[16] Chen H., Schall M.C., Fethke N. (2017). Effects of Movement Speed and Magnetic Disturbance on the Accuracy of Inertial Measurement Units. Proc. Hum. Factors Ergon. Soc. Annu. Meet.

[17] Aslani N., Noroozi S., Davenport P., Hartley R., Dupac M., Sewell P. (2018). Development of a 3D Workspace Shoulder Assessment Tool Incorporating Electromyography and an Inertial Measurement Unit—A Preliminary Study. Med. Biol. Eng. Comput..

[18] Tabrizi S.S., Pashazadeh S., Javani V. (2020). Data Acquired by a Single Object Sensor for the Detection and Quality Evaluation of Table Tennis Forehand Strokes. Data Brief.

[19] Punchihewa N.G., Miyazaki S., Chosa E., Yamako G. (2020). Efficacy of Inertial Measurement Units in the Evaluation of Trunk and Hand Kinematics in Baseball Hitting. Sensors.

[20] Roetenberg D., Veltink P. (2005). Camera-Marker and Inertial Sensor Fusion for Improved Motion Tracking. Gait Posture.

[21] Marković S., Kos A., Vuković V., Dopsaj M., Koropanovski N., Umek A. (2021). Use of IMU in Differential Analysis of the Reverse Punch Temporal Structure in Relation to the Achieved Maximal Hand Velocity. Sensors.

[22] Topley M., Richards J.G. (2020). A Comparison of Currently Available Optoelectronic Motion Capture Systems. J. Biomech..

[23] Huynh D.Q. (2009). Metrics for 3D Rotations: Comparison and Analysis. J. Math. Imaging Vis..

[24] Wu G., van der Helm F.C.T., (DirkJan) Veeger H.E.J., Makhsous M., Van Roy P., Anglin C., Nagels J., Karduna A.R., McQuade K., Wang X. (2005). ISB Recommendation on Definitions of Joint Coordinate Systems of Various Joints for the Reporting of Human Joint Motion—Part II: Shoulder, Elbow, Wrist and Hand. J. Biomech..

[25] Ong Z.C., Seet Y.C., Khoo S.Y., Noroozi S. (2018). Development of an Economic Wireless Human Motion Analysis Device for Quantitative Assessment of Human Body Joint. Measurement.

[26] Raghavendra P., Sachin M., Srinivas P.S., Talasila V., Vishwakarma H.R., Akashe S. (2017). Design and Development of a Real-Time, Low-Cost IMU Based Human Motion Capture System. Computing and Network Sustainability.

[27] Cottam D.S., Campbell A.C., Davey M.P.C., Kent P., Elliott B.C., Alderson J.A. (2022). Measurement of Uni-Planar and Sport Specific Trunk Motion Using Magneto-Inertial Measurement Units: The Concurrent Validity of Noraxon and Xsens Systems Relative to a Retro-Reflective System. Gait Posture.

[28] Sers R., Forrester S., Moss E., Ward S., Ma J., Zecca M. (2020). Validity of the Perception Neuron Inertial Motion Capture System for Upper Body Motion Analysis. Measurement.

[29] Robert-Lachaine X., Mecheri H., Muller A., Larue C., Plamondon A. (2020). Validation of a Low-Cost Inertial Motion Capture System for Whole-Body Motion Analysis. J. Biomech..

[30] Chia L., Andersen J.T., McKay M.J., Sullivan J., Megalaa T., Pappas E. (2021). Evaluating the Validity and Reliability of Inertial Measurement Units for Determining Knee and Trunk Kinematics during Athletic Landing and Cutting Movements. J. Electromyogr. Kinesiol..

[31] Robert-Lachaine X., Mecheri H., Larue C., Plamondon A. (2017). Validation of Inertial Measurement Units with an Optoelectronic System for Whole-Body Motion Analysis. Med. Biol. Eng. Comput..

[32] Caruso M., Sabatini A.M., Laidig D., Seel T., Knaflitz M., Della Croce U., Cereatti A. (2021). Analysis of the Accuracy of Ten Algorithms for Orientation Estimation Using Inertial and Magnetic Sensing under Optimal Conditions: One Size Does Not Fit All. Sensors.

[33] Valtonen Ornhag M., Persson P., Wadenback M., Astrom K., Heyden A. Trust Your IMU: Consequences of Ignoring the IMU Drift. Proceedings of the 2022 IEEE/CVF Conference on Computer Vision and Pattern Recognition Workshops (CVPRW).

[34] McAtamney L., Nigel Corlett E. (1993). RULA: A Survey Method for the Investigation of Work-Related Upper Limb Disorders. Appl. Ergon..

[35] Gilles M.A., Guélin J.-C., Desbrosses K., Wild P. (2017). Motor Adaptation Capacity as a Function of Age in Carrying out a Repetitive Assembly Task at Imposed Work Paces. Appl. Ergon..

[36] Gilles M.A., Wild P. (2018). Grasping an Object at Floor-Level: Is Movement Strategy a Matter of Age?. Appl. Ergon..

[37] Martin V., Reimann H., Schöner G. (2019). A Process Account of the Uncontrolled Manifold Structure of Joint Space Variance in Pointing Movements. Biol. Cybern..

[38] Rein R., Bril B., Nonaka T. (2013). Coordination Strategies Used in Stone Knapping. Am. J. Phys. Anthropol..

[39] Claudon L., Desbrosses K., Gilles M.A., Pichené-Houard A., Remy O., Wild P. (2020). Temporal Leeway: Can It Help to Reduce Biomechanical Load for Older Workers Performing Repetitive Light Assembly Tasks?. Appl. Ergon..

